# Nanotechnology in cancer therapeutics, diagnosis, and management

**DOI:** 10.5114/bta.2024.141807

**Published:** 2024-09-30

**Authors:** Disha Hazarika, Sumit Sarma, Priyanka Shankarishan

**Affiliations:** University of Science and Technology Meghalaya (USTM), Meghalaya, India

**Keywords:** cancer, diagnosis, management, nanotechnology, therapeutics

## Abstract

Nanotechnology presents an exciting opportunity in cancer research by offering significant advancements in therapies, diagnosis, and management. It possesses unparalleled potential to enhance the accuracy and effectiveness of cancer therapy while simultaneously reducing adverse effects, owing to its distinctive capability to manipulate matter at a molecular level. Using nanoparticle carriers has facilitated the precise administration of therapeutic agents to afflicted areas within the human body through customized drug delivery systems, resulting in improved treatment accuracy and efficacy while reducing adverse effects. These techniques improve drug solubility and stability, leading to elevated levels of biochemical availability and improved efficacy outcomes for patients with minimal negative effects during treatment cycles. Another use case for nanoparticles includes tumor imaging; functionalized with targeting ligands containing diagnostic agents, they foster early detection, making quicker remedial action plans possible. Overall, the incorporation of nanotechnology ensures a promising future, although it stresses the need to address regulatory hurdles and safety concerns before widespread clinical implementation. Despite the complexity of cancer research and patient care, nanotechnology shows promise in transforming both fields.

## Introduction

Cancer is a disease caused by the unbridled proliferation of abnormal cells that infiltrate neighboring tissues and organs, eventually metastasizing to distant parts of the body through the circulatory and lymphatic systems. Despite its localized onset, cancer can spread throughout the body via these systems. Its entanglement with various physiological cellular systems, including the inhibition of apoptosis in cell signaling, makes understanding this affliction challenging. The complex interactions between genetic and phenotypic levels, along with clinical variability and therapeutic resistance, necessitate a multi-faceted approach to treating cancer. Unfortunately, cancer remains a leading cause of mortality globally. By 2030, it is projected that the annual count of cancer-related fatalities and occurrences will escalate to 30 million (Lancet, 2018). Timely identification and management of malignant tumors are crucial strategies for reducing fatality rates and preventing the spread of the disease.

Traditional approaches for detecting cancer in its early stages typically involve the use of imaging modalities and the morphological examination of tissue (histopathology) or cells (cytology) (Gillies and Schabath, 2020). The standard methods for treating cancer are limited to chemotherapy, radiation, and surgery. However, contemporary cancer therapies often face challenges such as nonspecific systemic dispersion of anticancer drugs, inadequate drug concentrations within the tumor, and the inability to accurately assess therapeutic outcomes (Lorscheider et al., 2021). Unfortunately, the nondiscriminatory administration of anticancer agents causes various side effects, and their suboptimal delivery capacity often hinders them from achieving the desired results. Therefore, standard diagnostic techniques for detecting cancer are not highly sensitive and are only effective once the malignant cells have expanded significantly.

The detection of malignant tumors at an early stage has become increasingly difficult due to the inherent limitations of conventional diagnostic methods. Conventional clinical and therapeutic classification methods are inadequate in predicting successful therapy and patient outcomes. Nevertheless, the early detection and treatment of malignancies before metastasis, at the preinvasive stage, may not significantly impact illness management or enhance the likelihood of successful treatment (Lawrence et al., 2023). The development of therapeutic cancer medications aims to accomplish two fundamental objectives: augmented targeted selectivity and improved delivery efficiency.

Statistics on cancer incidence and prevalence show an urgent need for modern technology in cancer diagnosis and therapy. Nanotechnology is extensively used in cancer research today. Two areas where nanoparticle (NP) techniques are predicted to have substantial future influence include improved early screening and diagnosis, as well as therapy regimens that are more selectively taken up by tumor cells and have lower off-target toxicity (Patra et al., 2018). Nanotechnology, which involves the engineering and manufacture of materials using atomic and molecular components, is anticipated to benefit all areas of medicine, with oncology being the earliest and most notable beneficiary so far.

The advancement of nanotechnology has garnered significant interest recently for its potential to enhance the transport of anticancer drugs to tumor tissue while minimizing their distribution and toxicity in healthy tissue. This facilitates illness detection and lessens the severity of the disease. The goal of cancer nanotechnology is to describe how cellular and molecular elements interact with nanoscale devices in the detection and treatment of cancer. In vivo imaging and therapy are among the areas of nanotechnology that are progressing quickly (Yang et al., 2023). This advancement undoubtedly has profound implications for the treatment of individuals with cancer.

This review will focus on the recent documented improvements in cancer detection, imaging, medication, and management that show promising results in enhancing the prognosis of cancer patients. Additionally, the analysis will provide a brief account of the progress made in the field of nanotherapeutics and highlight the exceptional features of nanoparticles (NPs) that differentiate them from regular cancer therapies, making them ideal for managing different categories of cancer.

## Nanotechnology-based cancer therapy

Depending on the desired usage, working with materials at extremely small scales, ranging from a few nanometers to several hundred nanometers, is part of the study of nanotechnology (Peer et al., 2020). The distinct physicochemical properties of nanomaterials are notably similar to those of larger-scale equivalent materials. Nanotechnology’s unique 1–100 nanometer scale and high surface-to-volume ratios allow it to address current obstacles in cancer therapy. This method enables the simultaneous coupling of many functional molecules, such as antibodies, anticancer drugs, tumor-specific ligands, and imaging probes, with nanoscale devices (Jin et al., 2020). The evaluation and application of cancer nanotechnology indicate a significant advancement in cancer detection, diagnosis, and treatment. The promise of cancer nanotechnology lies in its capacity to design vehicles with special therapeutic qualities that can be highly selective and can effectively infiltrate tumors due to their small size.

## Tools of cancer nanotechnology

### Liposomes

**Fig. 1:**
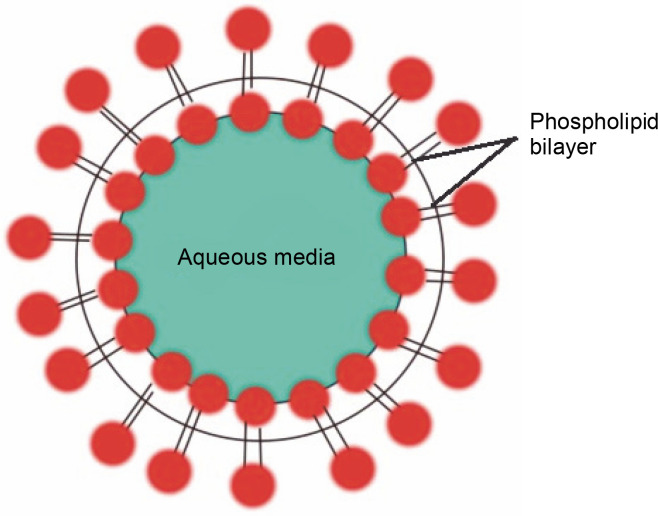
Liposome: lipoidal vesicles are used as drug carriers and are composed of bilayers of lipids entrapping an aqueous volume in the center

Liposomes are synthetic, tiny, spherically-shaped vesicles made of cholesterol and nontoxic phospholipids (Fig. 1). Anticancer medicines are encapsulated in liposomes to reduce their harmful effects and enhance their efficacy. Their primary method of operation involves the transportation of both hydrophilic and hydrophobic molecules. Due to their varied structures and contents, liposomes have become valuable tools in biology, medicine, and biochemistry. Examples of liposome-mediated drug delivery include liposomal encapsulated doxorubicin (Doxil) (Baetke et al., 2015; Zhang and Cheng, 2020; Pérez-Herrero and Fernández-Medarde, 2015), cisplatin, and daunorubicin (Daunoxome) (Nogueira et al., 2015). These first-generation nanocarriers are now licensed by the FDA for therapeutic purposes and are marketed as promising liposome delivery systems (Table 1).

**Table 1:** Tools of cancer nanotechnology, their usage, and applications

Nanotechnology tools	Size range [nm]	Usage and applications
Liposomes	40–180	It is used to deliver chemotherapeutic drugs that ensure a higher accumulation of drugs upon the solid tumors; used in breast cancer, neuroblastoma, gene transfer, etc.
Polymeric micelles	10–100	It can tolerate the maximum toxicity of the drug, also can handle any pH range; thus, good for mediating experimental drugs on cancer treatment.
Dendrimers	1–10	It has both diagnostic and therapeutic applications. It can be used as an antifungal material, for inflammation, helps in special cell binding, and is also used as a drug-delivering agent
Carbon nanotubes	0.8–2.4	It has antifungal activities, and provides the least physical harm to organs; thus, very useful for delivering drugs. It has also a potential application in tumor imaging
Quantum dots	2–10	This can be used in the detection of cancer cells, detection of microorganisms, DNA detection, and imaging of the disease

### Polymeric micelles

A group of amphiphilic surfactant molecules is called a micelle. Polymeric micelles are microscopic structures composed of a hydrophobic polymer core, such as propylene glycol, and polymer blocks, such as polyethylene glycol. These are solid micelles, ranging in size from 10 to 1000 nm (Kim et al., 2018). They are frequently employed in drug discovery as hydrophobic drug carriers (Wang et al., 2018; Shanmugam et al., 2020; Martinelli et al., 2021). It appears that micelles will play a major role in therapeutics in the future. Numerous formulations of polymeric PEG micelles are undergoing clinical trials. One such formulation, loaded with doxorubicin, underwent a phase 1 clinical trial for solid tumors and demonstrated promising outcomes in treating restenosis, a condition caused by excessive growth of scar tissue, by promoting accumulation in vascular lesions (Han et al., 2019).

### Nanocantilever

Nanocantilevers are nanoscale devices with mechanical sensors used to detect pathogens in a system. These tools can interact with biomolecules and are utilized for diagnosis, genome studies, and drug discovery related to various *in vivo* diseases, primarily cancer. Nanocantilevers are very small bars anchored at one end and engineered with such precision that they can bind to cancer-causing molecules at the other end. These biomolecules can attach themselves to various DNA proteins associated with specific types of cancer. During detection, the cantilever bends at the binding site as a result of the interaction between the biomolecules and the receptor, forming bonds (Okamura et al., 2016; Nasir et al., 2021). By observing this deflection, it is feasible to determine whether the joined cells are malignant. Early diagnosis is possible if malignant cells are detected. The amount of DNA or protein attached to the surface can be quantified by monitoring the deflection and frequency of the beams, which are directed toward the binding site using silicon beams for detection (Okamura et al., 2016).

### Dendrimers

**Fig. 2:**
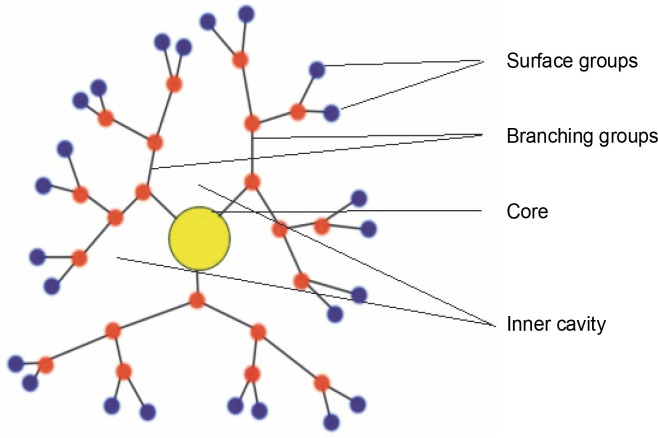
Dendrimers: hyperbranched nanocarrier comprising of the central core which can trap a huge amount of drug molecule protected by peripheral groups from being metabolized in the body

Dendrimers are artificial macromolecular polymers with recurrent branch-chain architectures, as shown in Figure 2. Due to their ability to change their internal size, shape, and arrangement, they are a valuable and complex drug delivery mechanism. Dendrimers can also serve as highly effective diagnostic tools for cancer imaging because of their special design, which allows for multivalent attachment for imaging probes (Wang et al., 2018; Kim et al. 2018). Because of their small size, dendrimers are employed in nanomedicine research. Additionally, some dendrimers have inherent cytotoxic and antibacterial properties, which give them medical applications of their own. Dendrimers frequently take on a three-dimensional spherical form. For example, the concentration of gadolinium-based magnetic resonance imaging contrast agents can be around 100 times lower than that of iodine atoms needed for computed tomography imaging, allowing them to be focused on a particular location and increasing imaging sensitivity. The first dendrimer pharmaceutical to gain recognition following clinical studies is Starpharma’s dendrimer-based microbicide, VivaGel, which is currently undergoing phase 1 clinical trials (Han et al., 2019).

### Carbon nanotubes

**Fig. 3:**
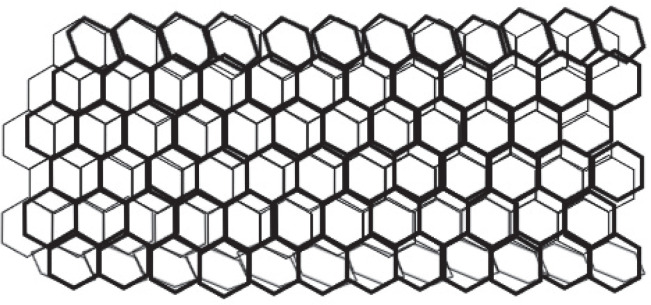
Carbon nanotubes: hollow cylindrical nanomaterial that can carry drugs inside and transport them throughout the body in a biocompatible way; the surface of CNTs is composed of a hexagonal arrangement of carbon atoms and is attached to chemical receptors that help in reaching the target cells

Carbon nanotubes (CNTs) are an additional class of nanodevice used for biomarker detection. CNTs are nanoscale carbon cylinders made of benzene rings (Fig. 3) and are used in biological research as sensors for DNA and protein detection. They also serve as drug, vaccine, or protein delivery vehicles and as diagnostic tools for differentiating between proteins obtained from serum samples. The investigation of the intriguing structural, mechanical, electrical, and optical properties of single-walled carbon nanotubes (SWNTs) for biological applications is an emerging field in nanotechnology. These applications include molecular transporters, drug-delivery biosensors, and the potential discovery of novel therapeutics. The high optical absorbance of SWNTs in the near-infrared zone under laser irradiation results in heating, which is useful for killing cancer cells specifically absorbed by the nanotubes. Currently, surface functionalization and the near-infrared fluorescence characteristics of SWNTs are the main areas of interest in bio-medical imaging. Surface-functionalized multiwalled carbon nanotubes have also been successfully employed for bio-mapping purposes. An *in vitro* study demonstrated that medications bonded to carbon nanotubes were more efficiently absorbed by the cells than free drugs alone (Okamura et al., 2016; Han et al., 2019; Nasir et al., 2021; Haung et al., 2021; Mohammadi et al., 2022).

### Quantum dots

**Fig. 4:**
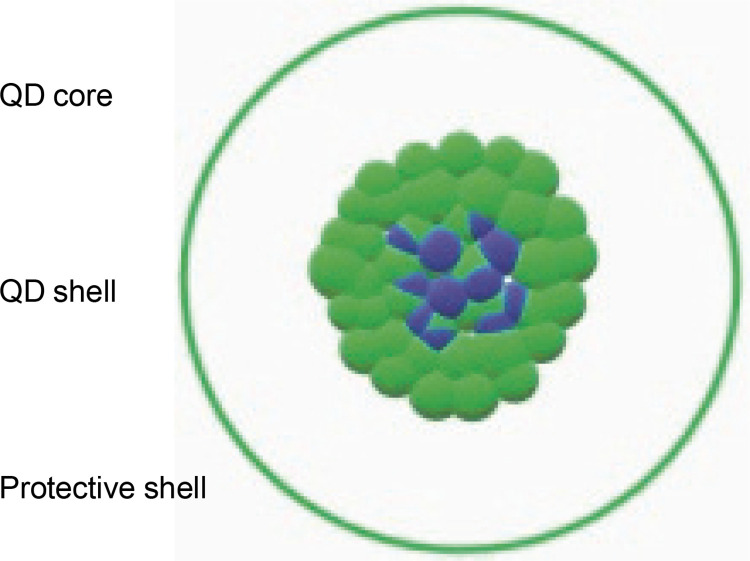
Quantum dots: semiconductor nanocrystals comprising of core (blue dots), shell (green dots), and protective shell (the hollow area inside a green circle); the core consists of semi-conductor material stabilized by a shell structure surrounding it

Quantum dots (QDs) are semiconductor NPs (Fig. 4) that have garnered significant interest from research organizations due to their scientific and technological significance in microelectronics, optoelectronics, and cell imaging (Zhang et al., 2019). Semiconductor QDs are emerging as a new type of fluorescent label for biology and medicine. QDs have broad absorption and narrow emission characteristics, enabling multicolor imaging with a single excitation source. Their unique physical, chemical, and optical properties, resistance to photo-bleaching, and high fluorescence quantum yield make QDs excellent choices for fluorescent targeting in *in vivo* molecular and cellular imaging (Nieland et al., 2019).

QDs hold great promise for *in vivo* and intraoperative tumor imaging primarily because of their intense fluorescent signals and multiplexing capabilities, which could enable high sensitivity and selectivity. Additionally, QDs offer a versatile nanoscale platform for designing multifunctional nanoparticles with both therapeutic and imaging functions. Despite their promising applications, QDs have come under toxicological scrutiny due to concerns about their material formulations. However, several groups have reported that the surface of QDs can be engineered or modified to improve their solubility, sensitivity, specificity, and visualization in target tissue using biocompatible surface coatings, such as PEG-silica (Zhang and Cheng, 2020; Mohammadi et al., 2022).

### NP-mediated drug delivery in cancer therapy

Despite advancements in fundamental cancer biology, current cancer treatment methods remain inefficient. Chemotherapy, one of the primary treatment options, often fails in clinical trials due to various factors such as the diverse genetic backgrounds of patients, ineffective drug delivery systems (DDSs) for single or combinatorial chemotherapy, and poor bioavailability of active agents. A significant limitation of conventional chemotherapy is that most anticancer agents cannot differentiate between cancerous and normal cells, leading to systemic toxicity and adverse effects by damaging healthy, rapidly dividing cells (Rodriguez and Zhao, 2012). Two basic requirements to make chemotherapeutic agents effective against cancer are: first, the ability to penetrate bodily barriers while maintaining volume and activity in the blood circulation; and second, the ability to specifically target tumor cells while sparing normal cells, releasing the drug in a controlled manner once it reaches the tumor site. Standard drug delivery methods, such as oral and intravenous (i.v.) administration, also have several drawbacks. Oral drugs require larger doses, which can increase toxicity or disrupt pharmacokinetics if exposed to the body’s metabolic pathways. Intravenous drugs often have low selectivity, resulting in negative effects on healthy tissues. Many medical researchers have turned to nanotechnology to address these therapeutic requirements.

Nanotechnology is being used in the development of smart drug delivery vehicles, also known as nanocarriers, for cancer therapeutic applications. NPs can carry a large number of therapeutic molecules and protect them from degradation, making them a potential medium for drug delivery. Nanocarriers have the potential to enhance the efficacy of drugs by improving the therapeutic ratio of currently available chemotherapeutics, controlling drug cytotoxicity based on the distribution profile of the NP (Elias et al., 2013; Maeda et al., 2013), and maintaining a steady therapeutic level of the drugs. They also aid in the development of multifunctional systems for targeted drug delivery, combination therapies, and simultaneous therapeutic and diagnostic applications.

The delivery of DOX (Doxorubicin) using liposomes, such as Doxil, is an effective example of nanotechnology-mediated medication delivery (Liu et al., 2013). This method has substantially reduced cardiotoxicity compared to free DOX. In 2005, the FDA approved the marketing of Abraxane®, an albumin-bound formulation of paclitaxel, which has proven to be promising and effective against breast and ovarian cancer (Zhang et al., 2013). Additionally, a non-PEGylated liposome (35–65 nm) called DaunoXome® is used in the treatment of AIDS-related Kaposi sarcoma (Fulton and Najahi-Missaoui, 2023). It contains DSPC (distearoylphosphatidylcholine) and cholesterol and has been widely studied in both pediatric and adult cancer patient populations before its approval. In recent years, several studies have proposed the use of nanocarriers or NPs as a novel approach for improved drug delivery. Table 2 shows selected anticancer nanomedicines approved for marketing and use by several countries.

**Table 2:** Anticancer nanomedicines: different pharmaceutical nanomedicines approved for use and their relative clinical trial stages

SL No.	Pharmaceutical nanocarrier	Product name	Company	Approved year	Active substance	Indication	Clinical trial stage	Reference
1	PEGylated liposomes	Zolsketil®	Accord Healthcare S.L.U.	2022	doxorubicin	breast cancer, ovarian cancer, multiple myeloma, and Kaposi’s sarcoma	phase III randomized study done received a marketing authorization valid throughout the European Union (EU)	Giordani et al. (2021)
2	dual-drug liposomal encapsulation	Vyxeos®	Jazz Pharmaceuticals Ireland Limited	2017 (FDA)	daunorubicin and cytarabine	acute myeloid leukemia	phase III trial was done ClinicalTrials.gov Identifier-NCT 01696084 authorized for use in the EU	Cortes et al. (2022)
3	PEGylated liposomal	ONIVYDE®	Les Laboratories Server	2015 (FDA)	irinotecan	metastatic adenocarcinoma of the pancreas	phase III clinical trial in metastatic pancreatic cancer done [NCT03088813] authorized for use in the EU	Pelzer et al. (2017), Tran et al. (2017)
4	non-PEGylated liposome doxorubicin (NPLD)	Myocet®	Elan Pharmaceuticals	2000 (EMEA)	doxorubicin	metastatic breast cancer	phase III randomized, controlled trial done [NCT00294996] authorized for use in the EU	Baselga et al. (2014)
5	polymeric micelle	Nanoxel ®	Fresenius Kabi India Pvt. Ltd.	2006 (India)	docetaxel	breast and ovarian cancers, AIDS-related Kaposi’s sarcoma and NSCLC	a phase III study done in non-muscle invasive bladder cancer [NCT02982395] phase IV study was done on breast cancer approved for use in India and several Asian and Latin American countries	Kim et al. (2018)
6	polymeric micelle-bound paclitaxel	Genexol-PM®	Lupin Ltd.	2007 (Korea)	paclitaxel	metastatic breast cancer, metastatic adenocarcinoma of the pancreas, non-small cell lung cancer (NSCLC)	phase III trial in metastatic HER2-negative breast cancer phase II clinical trial in metastatic adenocarcinoma of the pancreas [NCT02739633] phase II clinical trial in non-small cell lung cancer (NSCLC) [NCT01770795]	Park et al. (2017), Ahn et al. (2014), Lee et al. (2008, 2023)
7	radio enhancer nanosized product	Hensify (NBTXR3)	Nanobiotix	2019 (EMA)	radiotherapy	soft tissue sarcoma	phase II & III in adult soft tissue sarcoma [NCT0237 9845] approved for use in the EU	Bonvalot et al. (2019)
8	polymeric nanoparticles	Eligard ®	Tolmar Pharmaceuticals, Inc.	2002 (FDA)	leuprolide acetate	prostate cancer	phase IV interventional stu-dy in prostate cancer approved for use in the USA, Europe, and several Asian countries	Malek et al. (2022)
9	albumin-bound paclitaxel	Abraxane ®	American Biosciencem, Inc.	2005 (FDA, EMA)	paclitaxel	various cancers including metastatic and pancreatic cancers	phase I trial in pancreatic cancer [NCT02394535] approved for use in EU, USA and other countries	Koay et al. (2022)
10	nanoparticles of superparamagnetic iron oxide coated with amino silane	NanoTherm ®	MagForce AG	2013 (EMA) 2018 (FDA)	Fe2O3 nanoparticles	various types of cancer, including glioblastoma, prostate, and pancreatic cancers	interventional (clinical trial) in glioblastoma multiforme patients [NCT06271421]	Maier-Hauff et al. (2011)
11	non-pegylated liposomes	DaunoXome ®	NeXstar Pharmaceuticals	1996 (FDA)	daunorubicin	AIDS-related Kaposi’s sarcoma	phase III clinical trial in HIV-associated Kaposi's sarcoma [NCT00002093]	FDA (1996), Gill et al. (1995)
12	pegylated liposome	Doxil (Caelyx)	Ortho Biotech and Schering-Plough	1995 (FDA) 1996 (EMA)	doxorubicin	Kaposi’s sarcoma, ovarian cancer, and multiple myeloma	phase I clinical trial in advanced or refractory ovarian or breast cancer [NCT0556 7601]	Tejada et al. (2002)

Nanotechnology has emerged as a viable option for targeted cancer therapy, allowing medicines and other therapeutic compounds to be delivered selectively to cancer cells while minimizing negative effects on healthy tissue. NPs can be engineered with specific physical and chemical qualities that enable them to accumulate in tumors via passive targeting, taking advantage of the tumor microenvironment’s distinctive traits. Furthermore, NPs can be functionalized with targeting ligands to achieve active targeting and highly selective delivery to cancer cells.

Both active and passive targeting can be used separately or combined. Combining active and passive targeting can enhance nanoparticle delivery to cancer cells. For example, NPs can be designed to contain both targeting ligands and physical features that enable passive targeting. This combined approach can potentially improve the selectivity and efficacy of NP delivery while lowering the risk of negative effects.

The use of nanotechnology in targeted cancer therapy has the potential to revolutionize cancer treatment by increasing chemotherapy efficacy while decreasing adverse effects. Additionally, NPs can be tailored for cancer imaging, enabling early identification and diagnosis of cancer and tracking treatment response. However, more research is needed to optimize NP design and better understand the complex interactions between NPs and cancer cells within the tumor microenvironment.

### Active targeting

**Fig. 5:**
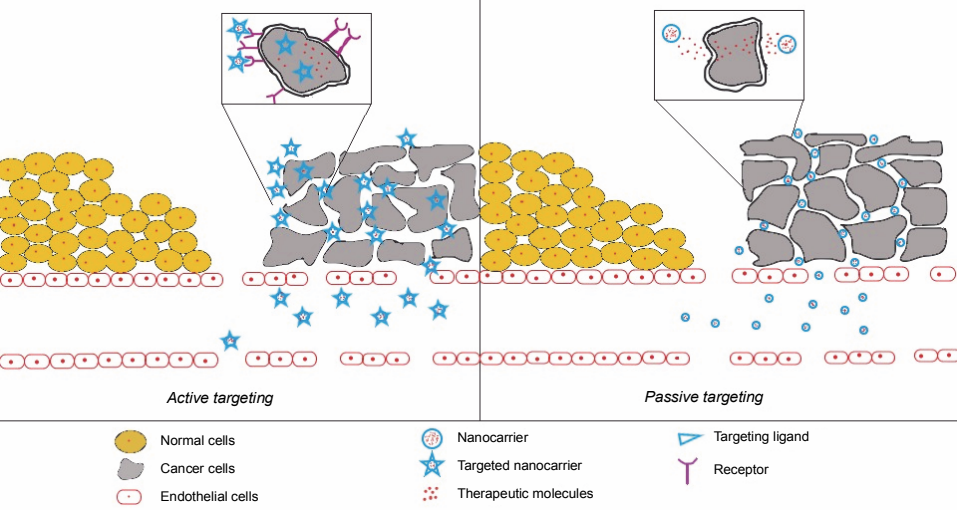
Schematic depiction of NPs mediated targeting of cancer cells; (A) active targeting is mediated by targeted NPs where the NPs conjugated with anticancer drugs are actively taken up by cancer cells after binding to their cell surface receptors on the cancer cells; (B) passive targeting with nanoparticles carrying anticancer drugs; here NPs passively concentrate in solid tumor tissue via an enhanced permeability and retention (EPR) effect leading to the local release of the drug near the cancer cells

Active targeting is a promising method of cancer treatment that utilizes nanotechnology. This method involves using targeting ligands on the surface of NPs that can specifically bind to cancer cells. These targeting ligands are usually peptides, antibodies, or aptamers that recognize and bind to cancer-specific receptors on the surfaces of cancer cells. Once the nanoparticles bind to the cancer cells, they can deliver their cargo, such as chemotherapeutic drugs, directly to the cancer cells, thereby improving chemotherapy efficacy and reducing side effects (Fig. 5).

One of the challenges in cancer treatment is the lack of specificity of chemotherapeutic agents, which can cause damage to healthy cells and tissues. Active targeting with NPs can enhance chemotherapy specificity by selectively delivering drugs to cancer cells (Pearce and O’Reilly, 2019). In this section, we will explore the concept of active targeting in cancer using nanotechnology and discuss some notable studies that support it.

The use of antibody-conjugated nanoparticles is one example of active targeting. Antibodies recognize and bind to their target antigens with high specificity, making them ideal candidates for active targeting. For instance, Mangadlao et al. (2018) demonstrated the successful targeting of prostate cancer cells using anti-PSMA (prostate-specific membrane antigen) antibody-conjugated gold NPs. The NPs bind to PSMA-expressing cancer cells, allowing for targeted delivery of chemotherapeutic drugs and improving therapeutic efficacy. In another study, Xiong et al. used folate-functionalized gold NPs to target cancer cells that overexpress folate receptors. The NPs were found to preferentially accumulate in tumor cells, resulting in increased therapeutic efficacy of the loaded drug and decreased toxicity to healthy tissues (Xiong et al., 2012).

Another type of targeting ligand that can be used for active targeting is peptides. Peptides are short chains of amino acids that can bind to cancer-specific receptors on the surface of cancer cells (Yang et al., 2014). For example, the RGD peptide can bind to integrins, which are overexpressed on the surface of many cancer cells, including those of breast, lung, and prostate cancers. RGD peptides can be conjugated to nanoparticles and used to deliver chemotherapeutic agents to cancer cells, resulting in increased efficacy and decreased toxicity (Wang et al., 2016).

Single-stranded nucleic acids that can bind to specific molecules are known as aptamers. The SELEX (systematic evolution of ligands by exponential enrichment) method is used to select aptamers, involving the iterative enrichment of a library of random nucleic acid sequences for sequences that bind to the target molecule. Aptamers can be chosen to specifically target cancer cells or cancer-specific molecules, enabling selective targeting of cancer cells. For example, aptamers can be selected to specifically target cancer-specific splice variants of CD44, a cell surface receptor overexpressed in many types of cancer cells, including breast, ovarian, and prostate cancers (Li et al., 2016). These aptamers can be conjugated to nanoparticles and used to deliver chemotherapeutic agents to cancer cells with greater efficacy (Liu et al., 2018).

Active targeting can also be used to deliver other therapeutic molecules, such as siRNA and miRNA, to cancer-specific genes or gene products, resulting in selective inhibition of cancer cell growth and survival (Halbur et al., 2019). For example, nanoparticles conjugated with siRNA targeting the oncogene Bcl-2 have been shown to selectively inhibit the growth of cancer cells overexpressing Bcl-2, resulting in improved cancer treatment efficacy (Rahman et al., 2018).

### Passive targeting

Passive cancer cell targeting with nanotechnology has become a viable strategy for improving cancer treatment efficacy. This method takes advantage of tumor tissues’ unique properties, such as poor lymphatic drainage and leaky vasculature. The enhanced permeability and retention (EPR) effect allows nanoscale particles to accumulate preferentially in tumor tissues, as depicted in Figure 5 (Chehelgerdi et al., 2023). This article examines the concept of passive targeting in cancer therapy, the various types of nanocarriers used for passive targeting, and the benefits and drawbacks of this approach.

The use of nanocarriers such as liposomes, polymeric NPs, and micelles, which can accumulate preferentially in tumor tissues due to the EPR effect, is central to passive targeting. The EPR effect occurs when tumor tissues have poor lymphatic drainage and leaky vasculature, allowing nanoscale particles to accumulate preferentially in the tumor tissue via passive diffusion and retention. This causes nanocarriers to accumulate selectively in tumor tissues while minimizing their distribution in healthy tissues. Compared to conventional chemotherapy, this selective accumulation of nanocarriers in tumor tissues results in improved drug efficacy and reduced toxicity (Jain, 2013).

Liposomes are among the most extensively researched nanocarriers for passive cancer targeting. They are spherical vesicles made up of a lipid bilayer capable of encapsulating both hydrophilic and hydrophobic drugs. Liposomes can accumulate in tumor tissues via the EPR effect and release the drug payload directly into the tumor tissue, reducing drug exposure to healthy tissues (Allen and Cullis, 2013). The FDA has approved several liposomal formulations for cancer therapy, including Doxil (doxorubicin hydrochloride liposome injection), with several others in clinical trials (Limi and Reikvam, 2023).

Another type of nanocarrier used for passive targeting in cancer therapy is polymeric NPs. Polymeric NPs, which can encapsulate both hydrophilic and hydrophobic drugs, are typically made of biodegradable polymers such as poly(lactic-co-glycolic acid) (PLGA). These NPs can accumulate in tumor tissues due to the EPR effect and gradually release the drug payload over time, resulting in long-term drug exposure in the tumor tissue (Kesharwani et al., 2019). Polymeric NPs have been shown to improve the efficacy of anticancer drugs like paclitaxel and cisplatin.

Micelles are another type of nanocarrier used for passive cancer targeting. Composed of amphiphilic molecules, micelles form spherical structures in aqueous solutions. The EPR effect allows micelles to encapsulate hydrophobic drugs and accumulate them in tumor tissues (Wang et al., 2018). The FDA has approved several micellar formulations for cancer therapy, including Genexol-PM (paclitaxel micellar), with several others in clinical trials.

The use of nanocarriers for passive targeting has several advantages over traditional chemotherapy. Firstly, the selective accumulation of nanocarriers in tumor tissues improves drug efficacy and reduces toxicity compared to conventional chemotherapy. Secondly, nanocarriers can encapsulate both hydrophilic and hydrophobic drugs, allowing for the simultaneous delivery of multiple drugs with different mechanisms of action, thereby enhancing cancer therapy efficacy. Thirdly, nanocarriers can shield the drug payload from degradation and clearance, resulting in long-term drug exposure in tumor tissue. Finally, nanocarriers can be functionalized by targeting ligands such as antibodies and peptides to improve cancer therapy selectivity and efficacy (Bazak et al., 2014).

Despite the benefits of passive targeting with nanocarriers, several challenges must be overcome before this approach can be widely used in clinical practice. One major challenge is tumor heterogeneity, which can affect the EPR effect and lead to variable nanocarrier accumulation in different regions of the tumor (Wilhelm et al., 2016). Another issue is the possibility of off-target effects, where nanocarriers can accumulate in healthy tissues with leaky vasculature, resulting in unintended toxicity. Furthermore, the complexity of the biological barriers that nanocarriers encounter during circulation, such as the mononuclear phagocyte system (MPS) and the endothelial barrier, can influence nanocarrier pharmacokinetics and biodistribution (Hrkach et al., 2012).

Passive targeting with nanocarriers is a promising approach for improving cancer therapy efficacy. Through the EPR effect, nanocarriers such as liposomes, polymeric NPs, and micelles can accumulate preferentially in tumor tissues, resulting in improved drug efficacy and reduced toxicity compared to conventional chemotherapy. However, several obstacles must be overcome before this approach can be widely used in clinical practice. Future research should focus on enhancing the design and formulation of nanocarriers to improve their selectivity, stability, and *in vivo* pharmacokinetics.

### NP-mediated gene therapy

DNA, the information carrier from generation to generation, is ideal for applications in the biomedical field due to its low cytotoxicity and high biocompatibility (Li et al., 2013; Chao et al., 2014; Linko et al., 2015; Surana et al., 2015; Chen et al., 2015). Nanotechnology leverages DNA to transfer and express genetic material into diseased cells for therapeutic applications. Recent studies have created artificial nanostructures using DNA, which are not present in biological systems (Li et al., 2013; Linko et al., 2015; Surana et al., 2015; Chen et al., 2015). DNA nanostructures are considered ideal drug delivery systems due to their exceptional molecular recognition properties, complementary base pairing, effective cellular internalization, and high and efficient drug loading capacity.

Various applications of DNA nanostructures include: 1) providing defined morphology to arrange organic, inorganic, and biomolecules by acting as scaffolds or templates; 2) serving as molecular transporters; 3) single-molecule spectroscopy; 4) acting as highly sensitive molecular and bio-detectors; 5) protein structure determination; 6) Vehicles for *in vitro* and *in vivo* drug delivery (Reif et al., 2012). The amphipathic property of DNA can be exploited to use single-stranded DNA to make carbon nanotubes (cNTs) suitable for in vivo use by solubilizing hydrophobic NPs. NPs prepared by binding 4 and 5 poly(amidoamine) (PAMAM) dendrimers and plasmid DNA have been confirmed to be effective for gene delivery (*in vitro* and *in vivo*) to colon and liver cancer cells.

Another technique, known as DNA origami, has been successfully used for nanopatterning nanoparticles, proteins, and other functional molecular components into well-defined arrangements, as well as for preparing different 2D and 3D nanostructures. A nanovector has been developed by PEGylating polyethyleneimine and binding the chlorotoxin (CTX) peptide in NPs. This nanovector is ligand-mediated and functionalized with an Alexa Fluor 647 near-infrared fluorophore. In recent years, the potential of augmenting gene therapy has been established by many researchers with the help of nanotechnology (Castro et al., 2012). This approach has addressed major issues related to antisense therapy, such as low transfection efficiency, DNA degradation, entry into diverse cell types, and toxicity of the transfecting agents, which has successfully translated into clinical trials (Yang et al., 2023).

### Cancer diagnosis and imaging using nanotechnology

Cancer is the leading cause of death worldwide, and diagnosing it remains one of modern medicine’s most difficult challenges. Traditional diagnostic methods, such as biopsies, CT scans, and MRIs, have limitations in terms of sensitivity and specificity, and they can be invasive, uncomfortable, or even harmful to the patient. Nanotechnology offers a promising solution to these limitations by enabling highly sensitive and specific cancer diagnosis and imaging with noninvasive and less harmful techniques (Dessale et al., 2022). In this overview, we will explore the most recent advancements in nanotechnology-based cancer diagnosis and imaging.

### Nanomaterials for cancer imaging

Nanomaterials have shown great promise in biomedical applications, particularly in cancer imaging. They offer several advantages over traditional imaging techniques, including increased sensitivity and specificity, improved contrast, and the ability to specifically target cancer cells. In this article, we will look at some of the most common nanomaterials used in cancer imaging, such as gold NPs, liposomes, quantum dots, carbon nanotubes, iron oxide, and polymeric NPs.

### Gold nanoparticles

Gold nanoparticles (AuNPs) have exceptional optical properties that make them ideal for cancer imaging (Hao et al., 2021). They exhibit high surface plasmon resonance (SPR) in the visible to near-infrared (NIR) range, making them highly absorbing and scattering (Sharma et al., 2021). AuNPs can be easily functionalized with biomolecules such as antibodies or peptides, allowing for precise cancer cell targeting (Gao et al., 2018). Additionally, they are biocompatible and nontoxic, making them suitable for *in vivo* imaging applications (Yu et al., 2021).

AuNPs have been used in various cancer imaging techniques, such as photoacoustic imaging (PAI), MRI, and CT (Zhao et al., 2020). In CT imaging, AuNPs act as contrast agents, increasing the contrast between cancerous and healthy tissue (Ma et al., 2021). In MRI, AuNPs can function as T1 or T2 contrast agents, enhancing the signal intensity of cancerous tissue (Cai et al., 2019). When illuminated with light, AuNPs act as absorbers, generating heat that produces an acoustic signal used to generate images of cancerous tissue (Huang et al., 2021).

### Quantum Dots

QDs are semiconductor nanocrystals with distinct optical and physical properties that make them appealing for biomedical applications, including cancer detection (Le and Kim, 2023). QDs have a high quantum yield, meaning they efficiently convert absorbed energy into fluorescence emission. Due to their broad excitation spectrum and narrow emission spectrum, they are suitable for multiplexed imaging (Zhao and Zeng, 2015). Additionally, QDs are resistant to photobleaching, making them ideal for long-term imaging applications.

Fluorescence imaging is one of the most common applications of QDs in cancer diagnosis. QDs can be conjugated to biomolecules like antibodies or peptides and targeted to cancer cells, enabling precise cancer cell imaging. They have been used to label and image cancer cells in vitro and in vivo in animal models. Moreover, QDs have been used in sentinel lymph node mapping, which involves injecting them near the tumor and tracking them to the lymph nodes to determine if the cancer has spread (Si et al., 2014).

Another potential application of QDs in cancer diagnosis is theranostics, which combines imaging and therapy in a single nanoparticle. QDs can be functionalized with drugs or therapeutic molecules and targeted to cancer cells, allowing for a more targeted approach to therapy (Zayed et al., 2019). Furthermore, the unique optical properties of QDs can be utilized in photodynamic therapy, which involves exposing QDs to light to generate reactive oxygen species and kill cancer cells.

Despite the potential benefits of using QDs in cancer diagnosis and therapy, there are also safety concerns. The heavy metal content in QDs can cause cytotoxicity, and their long-term effects on living organisms are still being studied. Additionally, the cost of mass-producing QDs may limit their availability for widespread clinical use.

### Magnetic nanoparticles

Magnetic nanoparticles (MNPs) are nanoscale particles with magnetic properties. Due to their distinct physicochemical properties and biocompatibility, they have emerged as promising candidates for cancer diagnosis. MNPs can be coated with various biocompatible materials to increase their stability and biocompatibility and can be surface-functionalized with specific biomolecules such as antibodies or peptides for targeted cancer imaging.

MRIs are among the most widely utilized imaging modalities for MNPs in cancer diagnosis. In MRI, MNPs serve as contrast agents, increasing the contrast between cancerous and healthy tissue (Wang et al., 2018). Due to their high magnetic moment, MNPs can generate significant signal amplification and improve the sensitivity and specificity of MRI. MNPs can be engineered to have various magnetic properties, such as superparamagnetism, ferromagnetism, and ferrimagnetism, which can be used to tailor imaging performance and optimize biodistribution *in vivo* (Kumar et al., 2021).

Additional imaging methods, such as magnetic particle imaging (MPI) and magnetic relaxometry (MRX), have also utilized MNPs. MPI is a cutting-edge imaging technique that uses MNPs as tracers, allowing for high-resolution, real-time, highly sensitive imaging with specificity. MRX is a noninvasive imaging technique that detects cancerous tissues by utilizing the relaxation properties of MNPs.

In addition to imaging, MNPs can be used for cancer therapy. Magnetic hyperthermia is a therapy that uses MNPs to generate heat and selectively kill cancer cells. When MNPs are exposed to an alternating magnetic field, heat is produced, causing hyperthermia and cell death in cancer cells (Li et al., 2021). MNPs that are selectively targeted to cancer cells can reduce the damage to healthy tissues and improve therapeutic outcomes.

### Organic nanoparticles

Several types of organic nanoparticles have been explored for cancer imaging applications. Here are some examples.

Liposomes are spherical vesicles formed by lipid bilayers. Due to their biocompatibility, versatility, and ability to encapsulate both hydrophobic and hydrophilic imaging agents, liposomes have been extensively studied for cancer imaging. They can be functionalized with targeting ligands such as antibodies or peptides to specifically bind to cancer cells, thereby improving imaging sensitivity and specificity (Wang et al., 2017).

Dendrimers are highly branched macromolecules with distinct structures. They have a high density of functional groups on their surface, which allows for the attachment of imaging agents and targeting ligands. Dendrimers can have controlled sizes and surface chemistries, making them suitable for a variety of imaging modalities such as fluorescence imaging, MRI, and nuclear imaging (Patri et al., 2015).

Polymeric NPs are made from synthetic or natural polymers. They can be fabricated with precise dimensions, shapes, and surface properties. Polymeric NPs can encapsulate imaging agents and have a longer circulation time in the bloodstream. They can also be surface-functionalized with targeting ligands to recognize specific cancer cells (Taratula et al., 2011).

Micelles are self-assembling structures formed by amphiphilic molecules in aqueous solutions. They consist of a hydrophobic core and a hydrophilic shell. Micelles can encapsulate hydrophobic imaging agents, enhancing their solubility and stability. Surface modifications can improve targeting and imaging efficacy in cancer cells.

Organic NPs can also be derived from natural sources such as proteins, peptides, or polysaccharides. These nanoparticles are biocompatible, biodegradable, and can be engineered to carry imaging agents. For example, albumin NPs and chitosan NPs have been investigated for cancer imaging applications (Li et al., 2015).

Biocompatibility, tunable properties, and the ability to incorporate targeting ligands for specific cancer cell recognition are all advantages of these organic NPs. Ongoing research focuses on improving their properties, increasing stability, and enhancing imaging capabilities to facilitate their translation into clinical applications for cancer diagnosis and imaging.

### Clinical applications of nanotechnology in cancer diagnosis and imaging

Nanotechnology has emerged as a powerful tool in cancer diagnosis and imaging, enabling early detection, therapy monitoring, image-guided surgery, personalized medicine, and accurate prognosis and staging. In this section, we will discuss the clinical applications of nanotechnology in cancer diagnosis and imaging.

Early cancer detection is critical for successful treatment and increased survival rates. NPs can assist in early cancer detection by enabling targeted imaging of small tumors that are difficult to detect with conventional imaging techniques (Biju, 2014). NPs can be engineered to accumulate in tumor tissues, allowing for highly sensitive and precise imaging of cancerous cells. For example, iron oxide NPs can be functionalized with specific targeting ligands that bind to tumor cells (Mahmoudi et al., 2011). These NPs can then be used as contrast agents in MRI to detect tumors at an early stage.

NPs can also be used to track the effectiveness of cancer therapy (Chen et al., 2019). The accumulation of nanoparticles in tumors over time can be observed using imaging methods like positron emission tomography (PET), CT, and MRI, providing information on treatment efficacy. This approach, known as molecular imaging, can provide clinicians with real-time information on tumor response to therapy, allowing them to adjust treatment regimens accordingly (Kircher et al., 2012). For instance, polymeric NPs can be loaded with chemotherapy drugs and functionalized with targeting ligands. These NPs can then be used to track drug accumulation in tumors over time, providing data on drug efficacy and toxicity. Furthermore, iron oxide NPs can be used as MRI contrast agents to monitor changes in tumor size and shape during treatment (Wabler et al., 2014).

NPs can also be used to guide tumor resection surgery. Fluorescence imaging and MRI can visualize NP accumulation in tumors, allowing for precise surgical resection of cancerous tissues. NPs can be functionalized with fluorescent dyes or radioisotopes to visualize tumor margins during surgery. This method, known as image-guided surgery, can improve tumor resection accuracy while reducing the risk of recurrence (Nagaya et al., 2017).

NPs can also be used to develop personalized cancer treatment approaches. Clinicians can create treatment regimens tailored to the genetic and molecular characteristics of individual tumors by using nanoparticles to deliver therapeutic agents to specific tumor cells. For example, NPs can be functionalized with nucleic acids like siRNA or miRNA, which can silence specific genes overexpressed in tumor cells. This method, known as RNA interference therapy (RNAi), enables the development of personalized cancer treatments targeting specific genetic mutations (Wang et al., 2011).

Another application of NPs is improving cancer prognosis and staging accuracy. Clinicians can learn about the size, location, and aggressiveness of cancerous tissues by visualizing the accumulation of nanoparticles in tumors. For instance, iron oxide NPs can be used as contrast agents in MRI to visualize lymph nodes and detect metastasis (Bonvin et al., 2017). Additionally, gold nanoparticles can be functionalized with specific targeting ligands to detect cancer-specific biomarkers and provide information on tumor aggressiveness.

### Nanotechnology-based biosensors employed in cancer management

Nanotechnology-based biosensors have emerged as a promising approach for cancer management due to their ability to sensitively and selectively detect cancerspecific biomarkers, monitor treatment response, and predict disease recurrence (Sharifianjazi et al. 2021). Commonly used nanomaterials in biosensor development include carbon nanotubes (CNTs), gold nanoparticles (AuNPs), magnetic nanoparticles (MNPs), and QDs (Laraib et al., 2022). These nanomaterials have distinct optical, electrical, and magnetic characteristics that enhance signal detection and transmission. They can be functionalized with targeting moieties that selectively bind to cancer-specific biomarkers, such as antibodies or aptamers, and conjugated with detection probes such as fluorescent dyes, MRI contrast agents, or electrochemical sensors.

Nanotechnology-based biosensors use a variety of sensing mechanisms to detect cancer biomarkers. Optical biosensors exploit the interaction of light with nanomaterials, resulting in changes in absorbance, fluorescence, or surface plasmon resonance (Malhotra and Ali, 2018). Electrochemical biosensors detect changes in electrical properties caused by biomolecule binding or interaction. Magnetic biosensors utilize the magnetic properties of nanomaterials for sensing purposes. For example, AuNPs can be functionalized with antibodies against cancer-specific biomarkers like PSA or HER2 and conjugated with detection probes to enable sensitive and selective detection of these biomarkers in blood or tissue samples (Huang et al., 2017). Magnetic NPs can be employed as MRI contrast agents for tumor imaging and monitoring treatment response. Additionally, bio-sensors can be integrated with drug delivery systems, allowing for real-time feedback and control of drug release (Cicha et al., 2022).

Despite the promising applications of nanotechnology-based biosensors in cancer management, several obstacles must be overcome before they can be extensively used in clinical settings (Zhang et al., 2019). These challenges include optimizing biosensor sensitivity and specificity, validating biosensor performance in clinical samples, and developing multianalyte detection biosensors (Prabowo et al., 2021). Future directions in cancer management using nanotechnology-based biosensors include the integration of biosensors with other technologies such as artificial intelligence and microfluidics, as well as the development of biosensors for point-of-care testing.

## Conclusion

Nanotechnology has transformed cancer therapy, diagnosis, and management by introducing new tools that provide more precise and effective treatments. Nanotechnology-based drug delivery techniques have increased treatment effectiveness by decreasing side effects and improving drug delivery to cancer cells. Furthermore, nanotechnology-based imaging techniques have enhanced cancer detection accuracy and sensitivity, allowing for earlier detection and treatment. Nanotechnology-based biosensors have also aided in the early detection of cancer biomarkers, improving cancer diagnosis and management. While challenges remain, nanotechnology holds enormous potential in cancer therapy, diagnosis, and management, and its continued development and application promise a brighter future in cancer treatment.
